# A comparative study on the antioxidant activity of methanolic extracts from different parts of *Morus alba* L. (Moraceae)

**DOI:** 10.1186/1756-0500-6-24

**Published:** 2013-01-19

**Authors:** Muhammad Ali Khan, Aziz Abdur Rahman, Shafiqul Islam, Proma Khandokhar, Shahnaj Parvin, Md Badrul Islam, Mosharrof Hossain, Mamunur Rashid, Golam Sadik, Shamima Nasrin, M Nurul Haque Mollah, AHM Khurshid Alam

**Affiliations:** 1 Department of Pharmacy, University of Rajshahi, Rajshahi, 6205, Bangladesh; 2 Department of Pharmaceutical and Biological Sciences, UCL School of Pharmacy, London, UK; 3 Senior Scientific Officer, BCSIR, Rajshahi, 6205, Bangladesh; 4 Department of Zoology, University of Rajshahi, Rajshahi, 6205, Bangladesh; 5 Department of Statistics, University of Rajshahi, Rajshahi, 6205, Bangladesh

**Keywords:** *Morus alba*, Moraceae, Oxidative stress, Antioxidant, Correlation and regression

## Abstract

**Background:**

Antioxidants play an important role to protect damage caused by oxidative stress (OS). Plants having phenolic contents are reported to possess antioxidant properties. The present study was designed to investigate the antioxidant properties and phenolic contents (total phenols, flavonoids, flavonols and proanthrocyanidins) of methanolic extracts from *Morus alba* (locally named as Tut and commonly known as white mulberry) stem barks (TSB), root bark (TRB), leaves (TL) and fruits (TF) to make a statistical correlation between phenolic contents and antioxidant potential.

**Methods:**

The antioxidant activities and phenolic contents of methanolic extractives were evaluated by *in vitro* standard method using spectrophotometer. The antioxidant activities were determined by total antioxidant capacity, DPPH (1,1-diphenyl-2-picrylhydrazine) radical scavenging assay, hydroxyl radical scavenging assay, ferrous reducing antioxidant capacity and lipid peroxidation inhibition assay methods.

**Results:**

Among the extracts, TSB showed the highest antioxidant activity followed by TRB, TF and TL. Based on DPPH and hydroxyl radical scavenging activity, the TSB extract was the most effective one with IC_50_ 37.75 and 58.90 μg/mL, followed by TRB, TF and TL with IC_50_ 40.20 and 102.03; 175.01 and 114.63 and 220.23 and 234.63 μg/mL, respectively. The TSB extract had the most potent inhibitory activity against lipid peroxidation with IC_50_ 145.31 μg/mL. In addition, the reducing capacity on ferrous ion was in the following order: TSB > TRB > TL > TF. The content of phenolics, flavonoids, flavonols and proanthocyanidins of TSB was found to be higher than other extractives.

**Conclusion:**

The results indicate high correlation and regression (*p-value <0 .001*) between phenolic contents and antioxidant potentials of the extracts, hence the Tut plant could serve as effective free radical inhibitor or scavenger which may be a good candidate for pharmaceutical plant-based products. However, further exploration is necessary for effective use in both modern and traditional system of medicines.

## Background

Oxidative stress (OS) is the imbalance between cellular production of reactive oxygen species (ROS) and the ability of cells to scavenge them. OS has been implicated as a potential contributor to the pathogenesis of several diseases, such as cancer, diabetes and heart disease [1]. ROS cause the damage of many cellular components including lipids, proteins and nucleic acids, such as DNA leading to subsequent cellular death by modes of necrosis or apoptosis [2]. The damage can become more widespread due to weakened cellular antioxidant defense systems. All biological systems have antioxidant defense mechanism that protects against oxidative damages and repairs enzymes to remove damaged molecules. However, this natural antioxidant mechanism can be inefficient, hence dietary intake of antioxidant compounds is important. Consumption of fruits and vegetables is known to lower the risk of several diseases, such as cancer, cardiovascular diseases and stroke caused by OS [3] and such health benefits are mainly imposed due to the presence of phytochemicals, such as polyphenols, carotenoids and vitamin E and C [4].

Although the phenolic compounds are commonly found in both edible and non edible herbs, cereals, fruits, vegetables, oils, spices and other plant materials [5,6], scientific information on antioxidant properties of endemic plants, limited to certain regions and known only by local populations, is still rather scarce. Therefore, the assessment of such properties remains an interesting and useful task, particularly to find new promising sources of natural antioxidants for functional foods and/or nutraceuticals [6,7].

*Morus alba* (locally known as Tut, commonly known as white mulberry, family: Moraceae) has been domesticated over thousands of years and adapted to the wide area of tropical, subtropical, and temperate zones of Asia, Europe, North and South America, Africa and India. It is extensively cultivated for leaf yield in sericulture [8]. Tut fruits contain phenolics and flavonoids contents, vitamin, fat (mainly linolic acid, palmitic acid, oleic acid) and minerals [9], and its leaves have fixed oil, carbohydrate, protein, tannin, alkaloids, sterol, flavonoids, glycosides and saponin [10,11]. Fruits, root and stem barks and leaves of Tut plant have been used in the treatment of inflammation, jaundice and hepatitis, cancer, diabetes, dislipidemia, diarrhoea, dyspepsia, edema, fever, headache, hypertension, purgative, anthelminthic and wounds [12-15]. Leaves of Tut plant have been reported to use in the treatment of depression, anxiety, cerebral ischemia, hepatic disease, cancer, diabetes, dislipidemia and ulcer [10,16-20]. However, there are only few reports on antioxidant activities of different parts of Tut plant. Therefore, in this study, we evaluated the comparative antioxidant activity of methanolic extractives from different parts of Tut plant and made a statistical correlation between phenolic contents and antioxidant activity.

## Methods

### Plant collection

Leaves, fruits, stem and root barks of Tut plant (Additional file 1: Figure S1) were collected from Rajshahi University campus, Rajshahi, Bangladesh, in May, 2011 and were identified by an expert taxonomist at the Department of Botany, University of Rajshahi. A voucher specimen was deposited to the herbarium in the Department of Botany, University of Rajshahi. Plant materials were then washed separately with fresh water to remove dirty materials and were shade dried for several days with occasional sun drying. The dried materials were ground into coarse powder by grinding machine and the materials were stored at room temperature for future use.

### Extract preparation

According to our initial assessment we found methanol as the best solvent for the extraction of Tut plant. Initially, we did extraction using several solvents including methanol, ethanol, dichloromethane and ethyl acetate and based on TLC behavior and amount of extract obtained/gm of material we chose methanol for extraction.

The extraction was performed according to Alam et al. [21]. About 500 gm of each powdered plant materials were taken in four amber colored reagent bottles and soaked the materials with 1.5 liter of methanol. The sealed bottles were kept for 15 days with occasional shaking and stirring. The final extracts were filtered seperately through cotton and then Whatman No.1 filter papers and was concentrated with a rotary evaporator under reduced pressure at 50°C to afford 30, 35, 45 and 40 gm extract of leaves, fruits, stem bark and root bark extract, respectively.

### Chemicals

1,1-diphenyl-2-picrylhydrazyl (DPPH), potassium ferricyanide, catechin (CA), ferrous ammonium sulphate, butylated hydroxytoluene (BHT), gallic acid (GA), ascorbic acid (AA), AlCl_3_, trichloro acetic acid (TCA), sodium phosphate, ammonium molybdate, tannic acid, quercetin, DMSO, EDTA, acetyl acetone and FeCl_3_ were purchased from Sigma Chemical Co. (St. Louis, MO, USA); potassium acetate, phosphate buffer, thiobarbituric acid were purchased from Sigma-Aldrich, USA; vanillin was obtained from BDH; folin-ciocalteus’s phenol reagent and sodium carbonate were obtained from Merck (Damstadt, Germany).

### Determination of total phenolics

Total phenolic contents in the extracts were determined by the modified Folin-Ciocalteu method described by Wolfe et al., 2003 [22]. An aliquot of the extract was mixed with 2 ml Folin-Ciocalteu reagent (previously diluted with water 1:10 v/v) and 2 ml (75 g/l) of sodium carbonate. The tubes were vortexed for 15 sec and allowed to stand for 20 min at 25°C for color development. Absorbance was then measured at 760 nm UV-spectrophotometer (Shimadzu, USA). Samples of extract were evaluated at a final concentration of 0.1 mg/mL. Total phenolic contents were expressed in terms of galic acid equivalent, GAE (standard curve equation: y = 0.0086x + 0.0105, R^2^ = 0.9997), mg of GA/g of dry extract.

### Determination of total flavonoids

Total flavonoids were estimated using method described by Ordon ez et al. [23]. To 0.5 ml of sample, 1.5 ml of methanol, 100 μl of 10% aluminum chloride, 100 μl of 1 M potassium acetate solution and 2.8 ml of distilled water was added. After one hour 30 minutes of incubation at room temperature (RT), the absorbance was measured at 420 nm. Extract samples were evaluated at a final concentration of 0.1 mg/mL. Total flavonoids content was expressed in terms of catechin equivalent, CAE (standard curve equation: y = 0.0135x + 0.0085, R^2^ = 0.9984), mg of CA/g of dry extract.

### Determination of total flavonols

Total flavonols in the plant extracts were estimated using the method of Kumaran and Karunakaran [24]. To 2.0 ml of sample (standard), 2.0 ml of 2% AlCl_3_ in ethanol and 3.0 ml sodium acetate (50 g/L) solutions were added. The absorption at 440 nm was read after 2.5 hours at 20°C. Extract samples were evaluated at a final concentration of 0.1 mg/mL. Total content of flavonols was expressed in terms of quercetin equivalent, QUE (standard curve equation: y = 0.0255x + 0.0069, R^2^ = 0.9995), mg of QU/g of dry extract.

### Determination of total proanthocyanidins

Determination of proanthocyanidins was based on the procedure reported by Sun et al. [25]. A volume of 0.5 ml of 0.1 mg/mL extract solution was mixed with 3 ml of 4% vanillin-methanol solution and 1.5 ml hydrochloric acid; the mixture was allowed to stand for 15 minutes. The absorbance was measured at 500 nm. Extract samples were evaluated at a final concentration of 0.1 mg/mL. Total content of proanthocyanidin was expressed in terms of catechin equivalent, CAE (standard curve equation: y = 0.567x − 0.024, R^2^ = 0.9801), mg of CA/g of dry extract.

### Determination of total antioxidant capacity

Total antioxidant capacity (TAC) of samples was determined by the method reported by Prieto et al. [26] with some modifications. 0.5 ml of samples/standard at different concentrations was mixed with 3 ml of reaction mixture containing 0.6 M sulphuric acid, 28 mM sodium phosphate and 1% ammonium molybdate into the test tubes. The test tubes were incubated at 95°C for 10 minutes to complete the reaction. The absorbance was measured at 695 nm using a spectrophotometer against blank after cooling at RT. Catechin was used as standard. A typical blank solution contained 3 ml of reaction mixture and the appropriate volume of the same solvent used for the samples/standard was incubated at 95°C for 10 minutes and the absorbance was measured at 695 nm. Increased absorbance of the reaction mixture indicated increase total antioxidant capacity.

### Ferrous reducing antioxidant capacity assay

The ferrous reducing antioxidant capacity (FRAC) of samples was evaluated by the method of Oyaizu [27]. 0.25 ml samples/standard solution at different concentration, 0.625 ml of potassium buffer (0.2 M) and 0.625 ml of 1% potassium ferricyanide, [K_3_Fe (CN)_6_ solution were added into the test tubes. The reaction mixture was incubated for 20 minutes at 50°C to complete the reaction. Then 0.625 ml of 10% trichloro acetic acid, TCA solution was added into the test tubes. The total mixture was centrifuged at 3000 rpm for 10 minutes. After which, 1.8 ml supernatant was withdrawn from the test tubes and was mixed with 1.8 ml of distilled water and 0.36 ml of 0.1% ferric chloride (FeCl_3_) solution. The absorbance of the solution was measured at 700 nm using a spectrophotometer against blank. A typical blank solution contained the same solution mixture without plant extracts/standard and it was incubated under the same conditions and the absorbance of the blank solution was measured at 700 nm. Increased absorbance of the reaction mixture indicated increase reducing capacity.

### DPPH radical scavenging assay

Free radical scavenging ability of the extracts was tested by DPPH radical scavenging assay (DRSA) as described by Choi et al. [28] and Desmarchelier et al. [29]. A solution of 0.1 mM DPPH in methanol was prepared and 2.4 ml of this solution was mixed with 1.6 ml of extract in methanol at different concentration. The reaction mixture was vortexed thoroughly and left in the dark at RT for 30 minutes. The absorbance of the mixture was measured spectrophotometrically at 517 nm. BHT was used as reference. Percentage DPPH radical scavenging activity (% DRSA) was calculated by the following equation,

(1) $$\left(\%\mathrm{DRSA}\right)=\left\{\left(A_{o}–A_{1}\right)/A_{o}\right\}\times 100$$

Where A_0_ is the absorbance of the control, and A_1_ is the absorbance of the extractives/standard.

Then % of inhibition was plotted against concentration, and from the graph IC_50_ was calculated.

### Hydroxyl radical scavenging activity

Hydroxyl radical scavenging activity (HRSA) of the extractives was determined by the method of Klein et al. [30] with a slight modification. 0.5 ml of extractives/standard at different concentration was taken in test tubes. 1 ml of Fe-EDTA solution (0.13% ferrous ammonium sulphate and 0.26% EDTA), 0.5 ml of 0.018% EDTA solution, 1 ml of 0.85% DMSO solution and 0.5 ml of 22% ascorbic acid were added into the test tubes. The test tubes were capped tightly and warm at 85°C for 15 minutes into the water bath. After incubation, the test tubes were uncapped and 0.5 ml ice cold TCA (17.5%) was added to each of test tubes immediately. 3 ml of nash reagent (7.5 gm of ammonium acetate, 300 μl glacial acetic acid and 200 μl acetyl acetone were mixed and made up to 100 ml) was added to all the tubes and incubated at RT for 15 minutes. Absorbance was taken in UV-spectrophotometer at 412 nm wave length. Percentage hydroxyl radical scavenging (% HRSA) activity was calculated using the following equation,

(2) $$\%\mathrm{HRSA}=\left\{\left(A_{o}–A_{1}\right)/A_{o}\right\}\times 100$$

Where A_0_ is the absorbance of the control, and A_1_ is the absorbance of the extractives/standard.

Then % of inhibition was plotted against concentration, and from the graph IC_50_ was calculated.

### Lipid peroxidation inhibition assay

The lipid peroxidation inhibition assay (LPI) was determined according to the method described by Liu et al. [31] with a slight modification. Excised rat liver was homogenized in buffer and then centrifuged to obtain liposome. 0.5 ml of supernatant, 100 μl 10 mM FeSO_4_, 100 μl 0.1 mM AA and 0.3 ml of extractives or standard at different concentration were mixed to make the final volume 1 ml. The reaction mixture was incubated at 37°C for 20 minutes. 1 ml of (28%) TCA and 1.5 ml of (1%) TBA was added immediately after heating. Finally, the reaction mixture was again heated at 100°C for 15 minutes and cool at RT. After cooling, the absorbance was taken at 532 nm. Percentage inhibition of lipid peroxidation (% LPI) was calculated by the following equation,

(3) $$\%\mathrm{LPI}=\left\{\left(A_{o}–A_{1}\right)/A_{o}\right\}\times 100$$

Where A_0_ is the absorbance of the control, and A_1_ is the absorbance of the extractives/standard.

Then % of inhibition was plotted against concentration, and from the graph IC_50_ was calculated.

### Statistical analysis

All analyses were carried out in triplicates. Data were presented as mean *±* SD. To evaluate significant relationships between experimental parameters by correlation and regression analysis, the *F-* and *t-tests* (p-value <0.001) were used. Free R-software version 2.15.1 ( http://www.r-project.org/) and Microsoft Excel 2007 (Roselle, IL, USA) were used for the statistical and graphical evaluations.

## Results

### Determination of TAC and FRAC

The TAC and FRAC of methanolic extractives of different parts of Tut plant were shown in Table 1. Methanolic extract of different parts of Tut plant showed considerable antioxidant activity compared to CA (standard). At the concentration of 100 μg/mL, the absorbance of methanolic extract of TL, TF, TSB, TRB and (+)-catechin were 0.148, 0.410, 0.684, 0.466 and 1.81, respectively; while at 400 μg/mL, the absorbance of methanolic extracts of TL, TF, TSB, TRB and (+)-catechin were 0.532, 0.916, 2.316, 1.690 and 3.875. The extractives were found to increase the total antioxidant activity with the increasing concentration of the extracts (Table 1).

**Table 1:** Absorbance of TAC and FRAC of different parts (TL, TF, TSB and TRB) of Tut. at different concentration

**Extractives**	**TAC**		**FRAC**	
	**At 100 μg/mL**	**At 400 μg/mL**	**At 100 μg/mL**	**At 400 μg/mL**
TL	0.148 ± 0.011^1^	0.532 ± 0.011	0.516 ± 0.027	1.152 ± 0.039
TF	0.410 ± 0.019	0.916 ± 0.016	0.088 ± 0.009	0.355 ± 0.013
TSB	0.684 ± 0.026	2.316 ± 0.031	0.555 ± 0.025	2.454 ± 0.193
TRB	0.466 ± 0.014	1.690 ± 0.017	0.659 ± 0.014	2.149 ± 0.064
AA	**-**	**-**	2.47 ± 0.008	3. 04 ± 0.163
CA	1.81 ± 0.041	3.875 ± 0.081	**-**	**-**

*NB*: ^1^Each value is the average of three analyses ± standard deviation. *TL* = Tut leaf, *TF* = Tut fruit, *TSB* = Tut stem bark, *TRB* = Tut root bark, *AA* = Ascorbic acid and *CA* = Catechin.

The methanolic extracts of four parts of Tut plant showed reducing activity, although less than that of ascorbic acid, a reference antioxidant, the extractives increased the reducing activity with the increased concentration of the extracts. At 100 μg/mL, the absorbance of methanolic extracts of TL, TF, TSB, TRB and ascorbic acid were 0.516, 0.088, 0.555, 0.659 and 2.47 respectively, while at 400 μg/mL, the absorbance of methanolic extracts of TL, TF, TSB, TRB and AA were 1.152, 0.355, 2.454, 2.149 and 3.04, respectively. A higher absorbance indicates a higher reducing power. These results demonstrated that the methanolic extracts of TSB and TRB had considerable iron reducing capacity.

### DPPH radical scavenging activity

**Figure 1:**
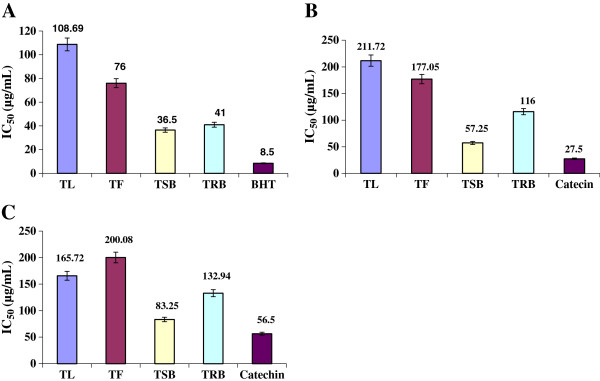
**Determination of IC**_**50 **_**of methanolic extractives from different parts of Tut plant (TSB, TRB, TL and TF): (A) DPPH assay (B) Hydroxyl radical scavenging assay and (C) Lipid peroxidation inhibition assay.** Data expressed as mean *±* SD (*n* = 3, *P <* .05) for all tested dosages.

Figure 1A shows the dose–response curve of DPPH radical scavenging activity of the methanolic extracts of TL, TF, TSB and TRB of Tut plant, compared with BHT. It was observed that the extract of TSB had higher activity than that of the other extractives. At a concentration of 100 μg/mL, the scavenging activity of the TL, TF, TSB and TRB reached 28.57, 62.75, 94.88 and 82.74%, while at the same concentration, that of the BHT was 96.354%. The IC_50_ of methanolic extracts of TL, TF, TSB and TRB were 108.69, 76.00, 36.50 and 41.00 μg/ml, respectively. The IC_50_ of BHT (standard) was 8.5 μg/mL (Figure 1A).

### Hydroxyl radical scavenging activity

The hydroxyl radical scavenging activity of the methanolic extracts of the TL, TF, TSB and TRB of Tut plant possess dose–response curve, compared with CA. It was observed that extract of the TSB had higher activity than that of the other extractives. At a concentration of 200 μg/mL, the scavenging activity of the TL, TF, TSB and TRB reached 47.23, 56.48, 81.10 and 74.21%, while at the same concentration, that of the CA was 81.07%. The hydroxyl radical scavenging activity of TSB of Tut plant was closely resembled to that of CA. The IC_50_ of methanolic extracts of TL, TF, TSB and TRB were 211.72, 177.05, 57.25 and 116.00 μg/mL, respectively. The IC_50_ of CA (standard) was 27.5 μg/mL (Figure 1B).

### Lipid peroxidation inhibition assay

The lipid peroxidation inhibition activity of the methanolic extracts of TL, TF, TSB and TRB of Tut plant was compared with CA. The methanolic extract of TSB had higher activity than that of the other extractives. At a concentration of 200 μg/mL, the scavenging activity of the TL, TF, TSB and TRB reached 30.67, 25.77, 61.51 and 43.12%, while at the same concentration, that of the catechin was 65.54%. The IC_50_ of methanolic extracts of TL, TF, TSB and TRB were 165.72, 200.08, 83.25 and 132.94 μg/mL, respectively. The IC_50_ of catechin (standard) was 56.5 μg/mL (Figure 1C).

### Total phenolic, flavonoids, flavonol and proanthocyanidin contents

**Figure 2:**
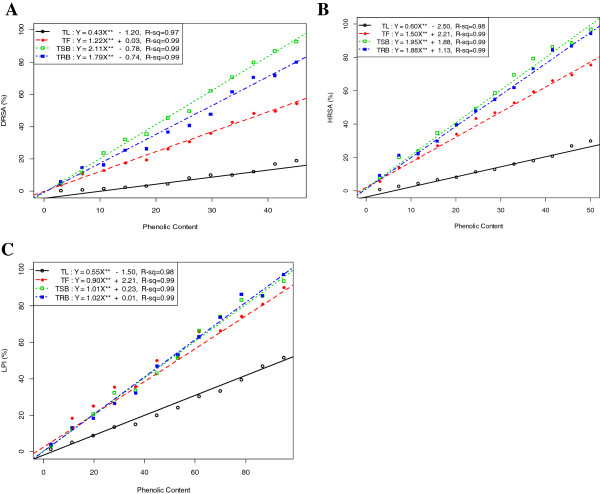
**Relationship of total phenolic contents with (A) % DRSA, (B) % HRSA and (C) % LPI.** Data expressed as mean *±* SD (*n* = 3, *P <* .001).

Table 2 shows the total polyphenols in the methanolic extract of TL, TF, TSB and TRB. Correlation of total phenolic contents of the extractives with free radical (DPPH^*·*^ and ^*·*^OH) scavenging efficiencies and %LPI are shown in Figure 2.

**Table 2:** Polyphenols content of the methanolic extracts of TL, TF, TSB and TRB

**Polyphenols**	**TL**	**TF**	**TSB**	**TRB**
Phenolics^*a*^	103.68 ± 17.47^1^	52.71 ± 3.17	285.62 ± 2.54	165.27 ± 3.28
Flavonoids^*b*^	6.667 ± 2.45	4.198 ± 2.26	102.469 ± 6.19	12.59 ± 2.96
Flavonols^*c*^	185.48 ± 1.19	149.01 ± 2.78	220.38 ± 1.26	132.54 ± 1.77
Proanthocyanidins^*b*^	2.36 ± 0.04	1.94 ± 0.25	4.68 ± 0.05	3.33 ± 0.07

*NB*: ^1^Each value is the average of three analyses ± standard deviation. *a*, *b* and *c* expressed in terms of GAE, CAE and QUE, respectively (mg of GA, CA and QU/g of dry extract, respectively).

### Correlation and regression of LPI with DRSA and HRSA

**Figure 3:**
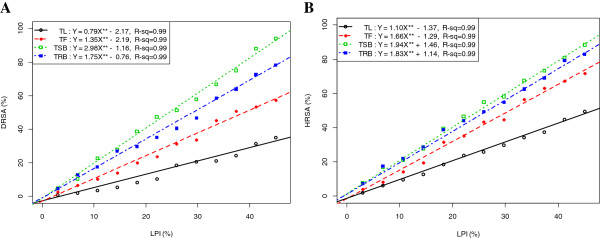
**Relationship of % LPI with (A) % DRSA and (B) % HRSA.** Data expressed as mean ± SD (*n* = 3, *P <* .001).

Figure 3 represents the correlation and regression (*p-value < 0.001*) of LPI with DRSA and HRSA. Significant correlations (*p-value < 0.001*) were observed for all the extractives (Figure 3).

## Discussion

### Total antioxidant property and ferrous reducing antioxidant property

The antioxidant potentials of the different parts of methanolic extracts of Tut plant were estimated from their ability to reduce the reduction of Mo (VI) to Mo (V) by the antioxidant compounds and subsequent formation of a green phosphate/Mo (V) complex at acidic pH. The reducing ability of the extractives was in the range of 0.532 ± 0.011– 2.316 ± 0.031 μm green phosphate/Mo (V) (Table 1). Antioxidant activity increased proportionally with the increase of polyphenols content. According to recent reports, a highly positive relationship between total phenols and antioxidant activity appears to be the trend in many plant species [32]. The iron reducing capacity of the methanolic extractives of TL, TF, TSB and TRB were estimated from their ability to reduce the Fe^3+^-ferricyanide complex to the ferrous form by donating an electron. The reducing ability of the extractives was in the range of 0.355 ± 0.013– 2.149 ± 0.064 μm Fe (II)/g (Table 1). In this study, ferrous reducing antioxidant capacity was increased with the increase of phenolic contents. Our results are consistent with the result published previously [33].

### DPPH radical scavenging activity

The effect of antioxidants on DPPH is thought to be due to their hydrogen donating ability [34]. Radical scavenging activities are very important to prevent the deleterious role of free radical in different diseases including cancer. DPPH free radical scavenging is an accepted mechanism by which antioxidants act to inhibit lipid peroxidation. This method has been used extensively to predict antioxidant activities because of the relatively short time required for analysis. Our results revealed that the methanolic extract of TSB had the similar free radical scavenging activity when compared with standard BHT (Figure 1A). The results indicated the proton-donating ability of the extractives which could serve as free radical inhibitors or scavengers and can also be served as primary antioxidants. The work performed adequately demonstrates that there exists correlation between polyphenolic contents of the extractives and its anti-oxidant properties. Consequently, this could be exploited as health care supplement [33].

### Hydroxyl radical scavenging activity

The mutagenic capacity of free radicals is due to the direct interactions of hydroxyl radicals with DNA and therefore playing an important role in cancer formation [35]. Hydroxyl radicals can be generated by biochemical reaction. Superoxide radical is converted by superoxide dismutase to hydrogen peroxide, which can subsequently produce extremely reactive hydroxyl radicals in the presence of divalent metal ions, such as iron and copper. The results demonstrated that the methanolic extract of four parts TL, TF, TSB and TRB had appreciable hydroxyl radical scavenging activity when compared with standard antioxidant, catechin (Figure 1B) and could be served as anticancer agent by inhibiting the interaction of hydroxyl radical with DNA. The ability of the extracts to quench hydroxyl radicals might directly relate to the prevention of lipid peroxidation.

### Lipid peroxidation inhibition assay

ROS induce membrane damage by peroxidising lipid moiety, specially the polyunsaturated fatty acids with a chain reaction known as lipid peroxidation [36]. The initial reaction generates a second radical, which in turn can react with a second macromolecule to continue the chain reaction leads to functional abnormalities of cells. In this study, lipid peroxidation of rat liver homogenates was induced by ferric ion plus ascorbic acid. Lipid peroxidation inhibition activity of TSB was found to be higher than other extractives (Figure 1C). These results indicated that Tut plant extracts have potential to be studied for use in treating liver disease.

### Total phenolic, flavonoids, flavonols and proanthocyanidin contents

Total phenolic contents of the extractives showed significant and strong positive correlation (*p-value <* .001) with free radical (DPPH^*·*^ and ^*·*^OH) scavenging efficiencies and %LPI (Figure 2). These results suggest a probable paramount role that the polyphenolic constituents of the extracts might play in free radical neutralization and lipid peroxidation inhibition.

### Correlation and regression of LPI with DRSA and HRSA

Significant correlations (*p-value < 0.001*) were observed for all the extractives for all dosages (Figure 3). This infers that the extractives differentially inhibit lipid peroxidation by virtue of their varying degrees of free radical quenching potential.

## Conclusion

The different parts of Tut plant have been used to treat a variety of diseases in Bangladesh as folk medicine. Compared to the effects of leaf, root and stem barks and ripe seeds on different diseases, little is known about the antioxidant activities of different parts of Tut plant. Our results clearly showed that the methanolic extract of TSB had strong hydroxyl and DPPH radical scavenging activities. The reducing capacity of TSB on ferrous ion was higher than that of other extractives. In addition, the potent antioxidative activity of Tut plant might result from its high contents of polyphenolic compounds. Hence, the methanolic extract from different parts of Tut plant could be used as a health-care food supplement and in the pharmaceutical industry.

## Abbreviations

AA: Ascorbic acid; CA: Catechin; CAE: Catechin equivalent; DPPH: 1,1-diphenyl-2-picrylhydrazine; DRSA: DPPH radical scavenging assay; FRAC: Ferrous reducing antioxidant capacity; GA: Gallic acid; GAE: Gallic acid equivalent; HRSA: Hydroxyl radical scavenging activity; LPI: Lipid peroxidation inhibition assay; OS: Oxidative stress; QU: Quercetin; QUE: Quercetin equivalent; ROS: Reactive oxygen species; RT: (Room Temperature); TAC: Total antioxidant capacity; TCA: Trichloro acetic acid; TF: Tut fruits; TL: Tut leaves; TRB: Tut root barks; TSB: Tut stem barks.

## Competing interests

The authors declare that they have no competing interests.

## Authors’ contributions

MAK: Designed the study and carried out the tests under the supervision of AHMKA. AAR: Helped to carry out the assay and draft the manuscript. SI: Carried out the lipid peroxidation inhibition assay. PK: Checked the grammatical errors and corrected the final manuscript. SP: Helped to carry out the assay. MBI: Helped to do the extraction. MH: Collected samples and provided solvent for extraction. MR: Helped to carry out the assay. GS: Helped to coordinate the biological assay. SN: Confirmed the antioxidant assay. MNHM: Performed statistical (correlation and regression) analysis. All authors read and approved the final manuscript.

## Supplementary Material

Additional file 1 **Figure S1.** Matured *Morus alba* L. (Moraceae) plant (locally known as Tut). Picture was taken on October, 2010 from botanical garden, Rajshahi University, Bangladesh. Click here for file
