# Supplementary material for: A comparative study on the antioxidant activity of methanolic extracts from different parts of Morus alba L. (Moraceae)
Source: BMC Res Notes. 2013 Jan 19;6:24. doi: 10.1186/1756-0500-6-24 (PMC3559264; doi:10.1186/1756-0500-6-24)
Supplement: Additional file 1 — Figure S1. Matured Morus alba L. (Moraceae) plant (locally known as Tut). Picture was taken on October, 2010 from botanical garden, Rajshahi University, Bangladesh. [file 1756-0500-6-24-S1.pdf]

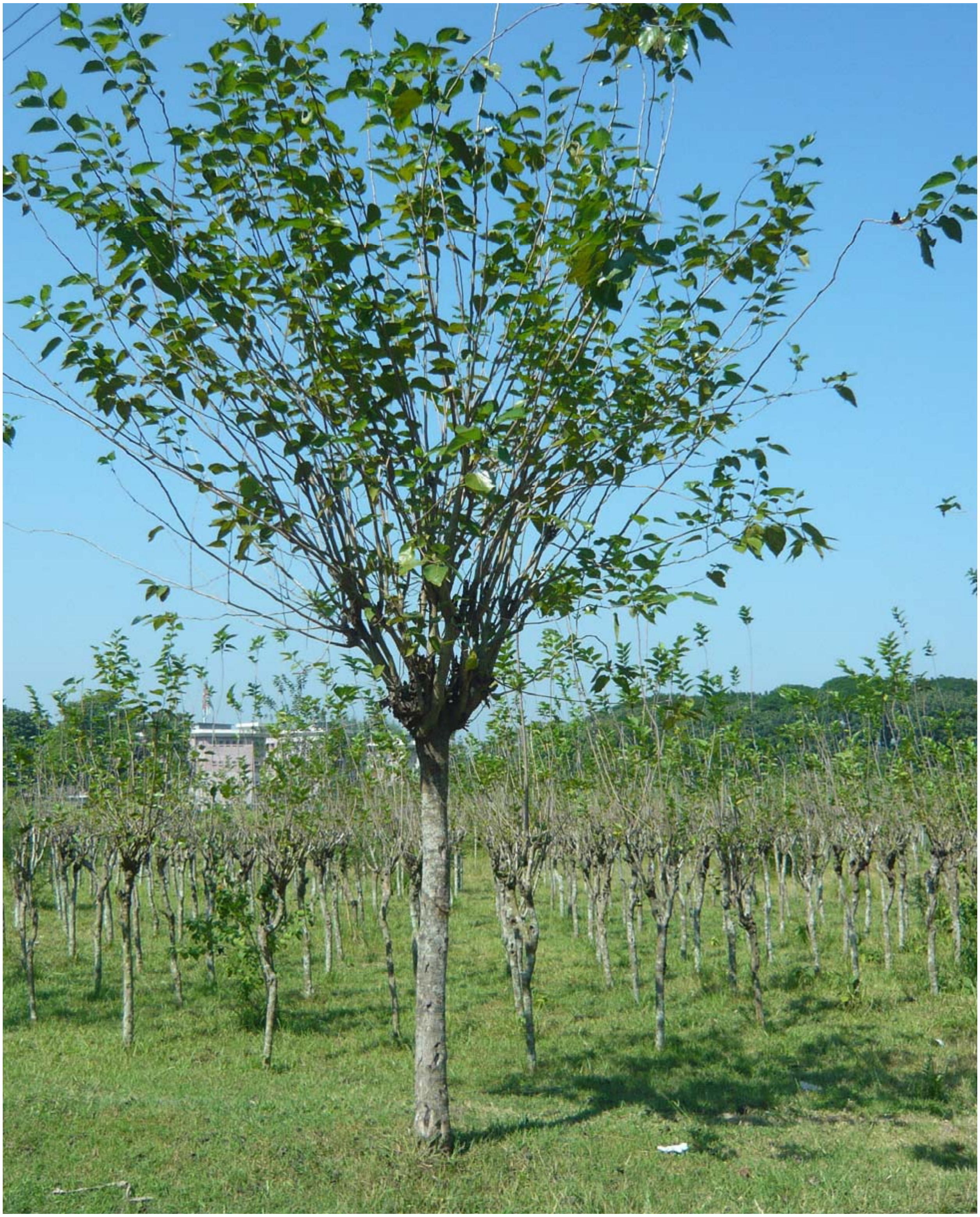

Supplementary Fig 1: Matured *Morus alba* L. (Moraceae) plant (locally known as Tut). Picture was taken on October, 2010 from botanical garden, Rajshahi University, Bangladesh.
